# Cognitive Training among Cognitively Impaired Older Adults: A Feasibility Study Assessing the Potential Improvement in Balance

**DOI:** 10.3389/fpubh.2016.00219

**Published:** 2016-10-17

**Authors:** Renae L. Smith-Ray, Cheryl Irmiter, Kristin Boulter

**Affiliations:** ^1^Department of Health Analytics, Research, and Reporting, Walgreen Co., Deerfield, IL, USA; ^2^Institute for Health Research and Policy, University of Illinois at Chicago, Chicago, IL, USA; ^3^Loyola University Chicago, Chicago, IL, USA

**Keywords:** dementia, cognitive training intervention, falls and fall risk prevention, older adults, quality of life

## Abstract

**Background:**

Emerging literature suggests that mobility and cognition are linked. Epidemiological data support a negative association between cognition and falls among cognitively intact older adults. A small number of intervention studies found that regimented cognitive training (CT) improves mobility among this population, suggesting that CT may be an under-explored approach toward reducing falls. To date, no studies have examined the impact of CT on balance among those who are cognitively impaired. The purpose of this study was to assess the feasibility of implementing a CT program among cognitively impaired older adults and examine whether there are potential improvements in balance following CT.

**Method:**

A single group repeated measures design was used to identify change in balance, depressive symptoms, and global cognition. A mixed method approach was employed to evaluate the feasibility of a CT intervention among a cohort of cognitively impaired older adults. CT was delivered in a group 2 days/week over 10 weeks using an online brain exercise program, Posit Science Brain HQ (20 h). All participants completed a one-on-one data collection interview at baseline and post-program.

**Results:**

Participants (*N* = 20) were on average 80.5 years old and had mild to moderate cognitive impairment. Following the 10-week CT intervention, mean scores on 4 of the 5 balance measures improved among CT participants. Although none of the balance improvements reached significance, these findings are promising given the small sample size. Depressive symptoms significantly improved between baseline and 10 weeks (*p* = 0.021). Mean global cognition also improved across the study period, but neither of these improvements were statistically significant. Based on participant responses, the CT program was feasible for this population.

**Conclusion:**

This study provides support for the feasibility of implementing a CT program among cognitively impaired older adults in an adult day setting. Our findings also add to emerging literature that CT may be a novel and innovative approach to fall prevention among older adults.

## Introduction

Each year approximately one-third of community-dwelling older adults fall (1). Falls increase with age, beginning at around 65, and approximately doubling by age 75 (2). Although numerous fall prevention interventions have been developed, the prevalence of falls is increasing (3, 4). A recent analysis of longitudinal Health and Retirement Study data found that the prevalence of falls increased from 28.2 to 36.3% between 1998 and 2010 (4). Currently, there is consensus that both intrinsic and extrinsic factors contribute to fall risk (5, 6). Intrinsic risk factors include: advanced age, female gender, white race, poor balance and gait, vestibular dysfunction, poor lower extremity strength, low vision, cardiovascular disease, depression, dementia, and cognitive decline. Extrinsic risk factors are polypharmacy, home environment, such as poor lighting, loose rugs, and footwear.

### Prevalence of Falls

A 2012 Cochrane Review of 159 fall prevention interventions implemented in community settings concluded that the most promising strategies for reducing falls risk are multifactorial and include multiple-component exercise programs, home safety assessment and modification, cardiac pacemakers when medically justified, and reduction of psychotropic medications (1). In 2015, the CDC identified 41 effective fall prevention interventions categorized into single-component interventions (exercise, home modification, and clinical) vs. multifactorial interventions (7). Of the 41, two multifactorial programs screen for cognitive decline, but do not intervene on this factor, and a third engages participants in dual-task processing (walking an obstacle course while listening to a story) as part of the intervention. To date, although many programs and resources have been allocated toward fall prevention, little, or no attention has been paid to cognitive factors; thus, there is an urgent need to examine cognitive interventions as a novel strategy for reducing falls incidence.

Cognitive processing specifically, executive function (EF), is linked to balance, gait, and falls (8–10). EF is characterized by three separate domains: (1) shifting, which includes attention, task switching, and dual-task processing; (2) updating, which involves updating working memory (WM) processes and representations; and (3) inhibition, related to decision-making, which involves inhibiting dominant and automatic responses (11–13). Speed of processing is a top-down process that impacts each of the three domains (14).

### Cognitive Training

As individuals progress into older age, fluid cognitive abilities, including EF, typically decline. Despite the decline in cognitive processing associated with increasing age, a growing body of evidence supports that the aging brain has exceptional neuroplasticity (15). Cognitive training (CT) involves completing tasks or exercises targeted toward a specific cognitive domain to promote neurogenesis within that domain (16, 17). CT that targets EF has been shown to be efficacious in maintaining or improving auditory speed, auditory accuracy, speed of processing, and mobility functions (18–20). A meta-analysis of 31 RCTs that included 1806 participants found that, compared to attention controls, CT significantly improved EF (21–23). This meta-analysis also showed that 9 of 10 interventions that examined maintenance of CT between 3 and 6 months found support for sustained training effects (24).

A limited number of studies have examined whether balance improves following CT (25–28). Two studies by Smith-Ray et al. found that balance improved among cognitively intact older adults following a 10-week group-based CT program. The first of these (25) involved participants (*N* = 51) who were older adult independent living (IL) residents with a history of falls. Participants were randomly assigned to a computer-based CT intervention (Posit Science) that met 3 days/week for 60 min over 10 weeks or to a no-contact control group that received CDC pamphlets on fall prevention. Individuals randomly assigned to the CT intervention demonstrated significantly better balance compared to controls (25). The second study assessed the feasibility of delivering the same CT intervention in community-based settings and examined its impact on balance and gait in community-dwelling black older adults with a history of falls or balance instability (*N* = 45). Participants were randomly assigned to CT or a no-contact control, but this time CT classes were held at Chicago Senior Centers over 10 weeks. Compared to controls, intervention participants improved significantly in balance and gait speed. Li et al. also used a randomized trial among older adults to show that CT was associated with significant improvements in body sway and dynamic balance compared to controls (28).

While, a growing body of literature supports the positive impact CT plays on cognition and mobility among healthy older adults, there is a gap in the literature on whether CT positively impacts balance among those who are cognitively impaired. Clinically recognized cognitive impairments, including dementia, can have a devastating impact on older adults’ memory, mood, quality of life (QOL), and behavior. Alzheimer’s disease, a common form of dementia, is estimated to affect over 5 million adults in the United States and rise to be between 11 and 16 million by the year 2050 (29). Alzheimer’s disease is the sixth leading cause of death and is the only one of the six causes that cannot be prevented or have progression slowed. Dementia, including Alzheimer’s disease, is generally associated with executive dysfunction which can contribute to instability in gait and balance (30). Individuals with Alzheimer’s disease are twice as likely to fall compared to the healthy older adults (31). People suffering from dementia often experience diminished health-related QOL due to various disease-related impairments, such as mobility and depression, which are also associated with cognitive decline (1). CT has been shown to improve depressive symptoms and health-related QOL among healthy older adults (6, 7). While, current literature has analyzed the impact physical activity has on improving EF and subsequent fall risk in cognitively impaired older adults, there are no studies assessing the impact CT has within this population. Therefore, the primary purpose of this study was to examine the feasibility of conducting group-based CT among a group of cognitively impaired older adults. The secondary purpose was to examine whether balance improved within this cohort following a 10-week CT intervention.

## Materials and Methods

### Design

A single group repeated measures design was used to identify change in balance, depressive symptoms, and global cognition. A mixed method approach was employed to evaluate the feasibility of a CT intervention among a cohort of cognitively impaired older adults. This evaluation was carried out in accordance with the University of Illinois Chicago, Office of the Protection of Research Subjects as an exempt status. All participant information was secondary data and de-identified before evaluation.

### Participants

Twenty participants were recruited from the Easter Seals adult day care program to participate in an online brain exercise program. All participants provided consent to participate. To be included in the study participants must have (1) been admitted to Easter Seals day services ≥1 month prior to program onset, (2) been fluent in English, (3) been able to engage in computerized cognitive tasks, (4) been willing to commit to the time commitments required by the program, (5) been cognitively impaired as indicated based on a score <27 on the mini mental state exam (MMSE), and (6) self-reported a diagnosis of mild cognitive impairment or early stage Alzheimer’s disease. Individuals were excluded if they (1) reported a physical impairment that would prohibit them from using a computer mouse or keyboard, (2) were unable to perform the neuropsychological evaluations, or (3) could not comprehend study instructions or were incapable of providing written informed consent.

### Procedure

Individual- and site-level data were gathered at baseline and post-intervention. CT was delivered in a group 2 days/week over 10 weeks using an online brain exercise program, Posit Science Brain HQ (20 h). Participants worked individually on desktop computers during weeks 2 through 9 to complete the CT. The Posit Science program targets EF through selective and divided attention, visuospatial WM, speed of processing, and dual-task processing – cognitive processes that are linked to balance and gait (9, 32, 33). The Posit Science Brain HQ training comprises 25 exercises that target a range of cognitive functions. Training through the Brain HQ program can be tailored to include a subset of exercises that target specific cognitive functions of interest. We tailored the CT intervention to include six Brain HQ exercises that target EF, including attention, visuospatial WM, inhibition, dual-task ability, and speed of processing.

### Measures

All participants completed a one-on-one data collection interview at baseline and post-program, week 10. Balance was measured by the 4-position balance stand and timed up and go (TUG). The 4-position balance stand consists of the most sensitive balance stands taken from the Berg Balance scale, a valid, reliable, and clinically relevant measure of balance in older adults. The TUG is a brief physical performance test that measures time required to stand up from chair, walk 3 m, turn around, and sit back down. The TUG is a valid and reliable measure of balance (34–36). Depression was measured using the geriatric depression scale (GDS). Cognition was measured using the MMSE and the cognitive self-report questionnaire (CSRQ).

In order to capture program feasibility, qualitative data were collected using key informant interviews with program staff to examine facilitators and barriers to program implementation and maintenance using the RE-AIM model.

### Analysis

Repeated measures paired *t*-tests were used to assess whether there were significant differences between the two time points. Given the pilot nature of this study, potential confounding factors were not controlled for in the analysis. All analyses were conducted using the IBM SPSS Statistics v 24.0 software.

## Results

**Figure 1:**
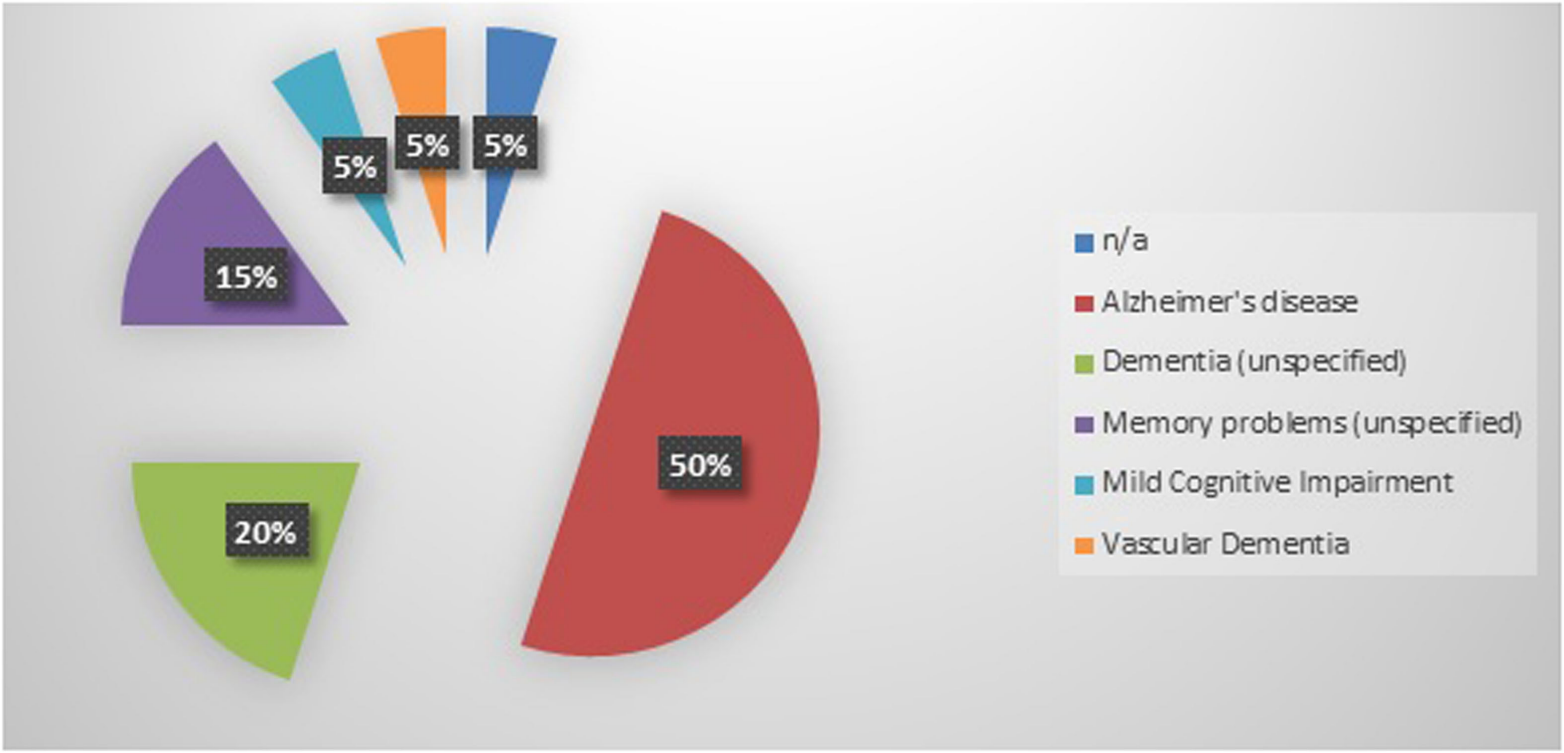
**Dementia diagnosis (*N* = 20)**.

Twenty cognitively impaired older adults were enrolled into this study. Participants had a mean age of 80.5 years and were categorized as having mild to moderate dementia as indicated by the mean baseline MMSE score of 21.4 (Table 1). The most frequently reported diagnosis of cognitive impairment was Alzheimer’s disease (50%), followed by unspecified dementia (20%), unspecified memory problems (15%), mild cognitive impairment (5%), and vascular dementia (5%) (Figure 1). A diagnosis of cognitive impairment was not available for one participant; however, the participant self-reported a diagnosis of clinically significant cognitive impairment.

**Table 1:** **Participant characteristics at baseline**.

		Frequency	Percent	Mean	SD
*N*		20	100		
Mean age		20	–	80.5	6.3
Mean MMSE		17	–	21.4	2.9
Marital status	n/a	1	5		
	Married	6	30		
	Single	1	5		
	Widowed	12	60		
Self-rated health	Excellent	1	5		
	Very good	6	30		
	Good	13	65		
	Fair	0	0		
	Poor	0	0		
MMSE cognitive impairment category	n/a	3	15		
	None/limited	5	25		
	Mild	11	55		
	Severe	1	5		

**Figure 2:**
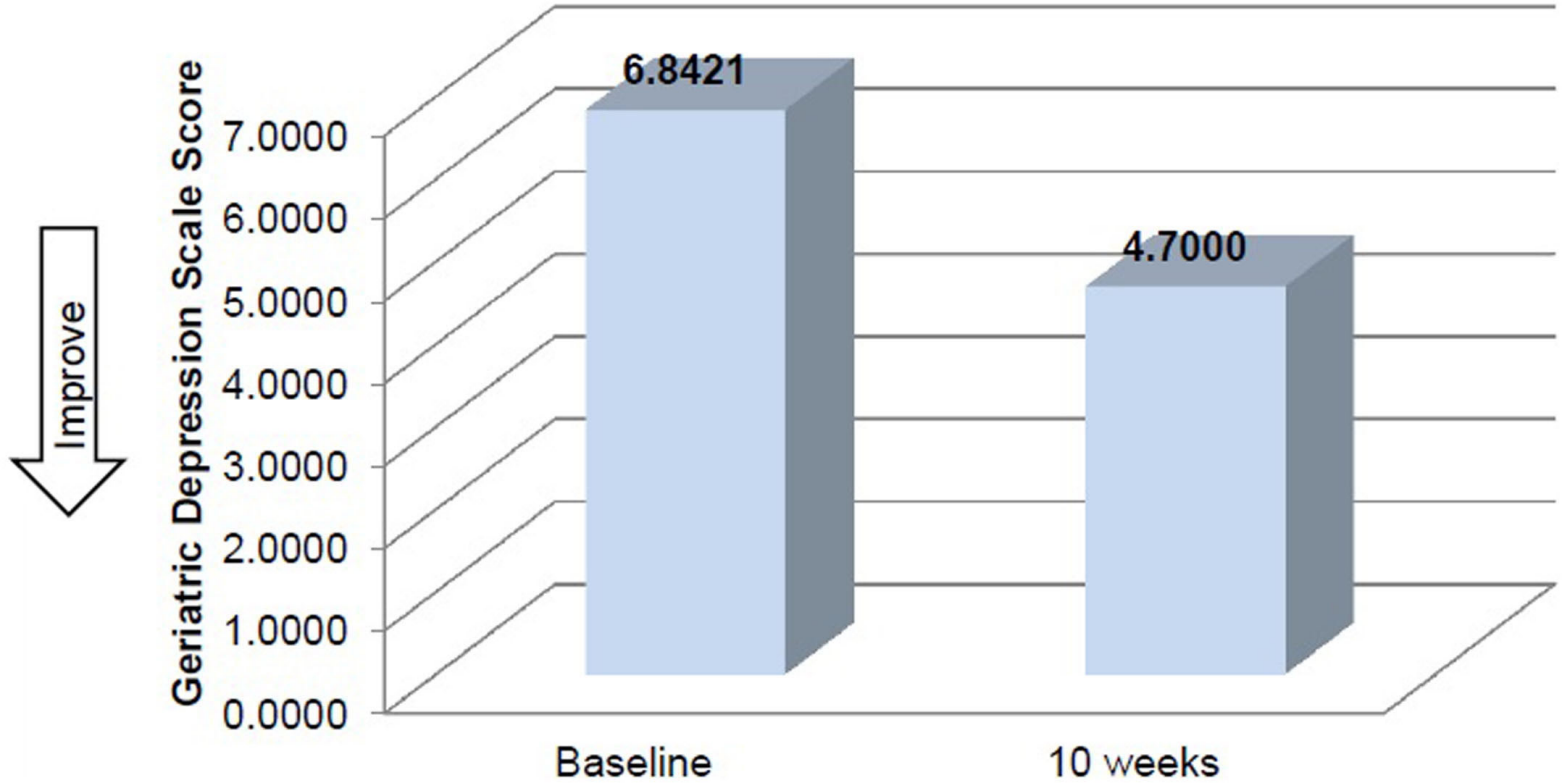
**Change in depressive symptoms between baseline and 10 weeks**.

**Figure 3:**
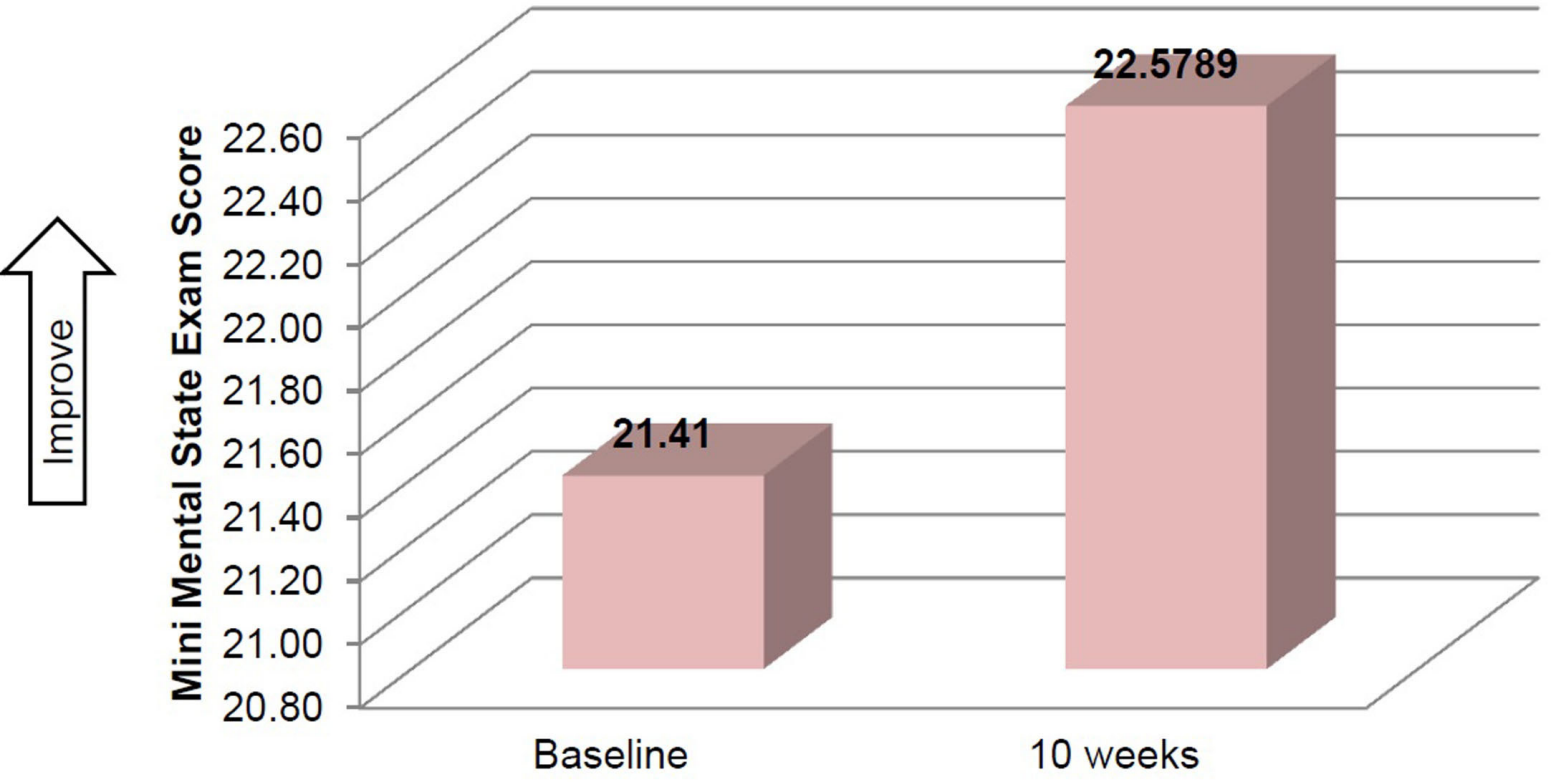
**Change in mini mental state exam between baseline and 10 weeks**.

**Figure 4:**
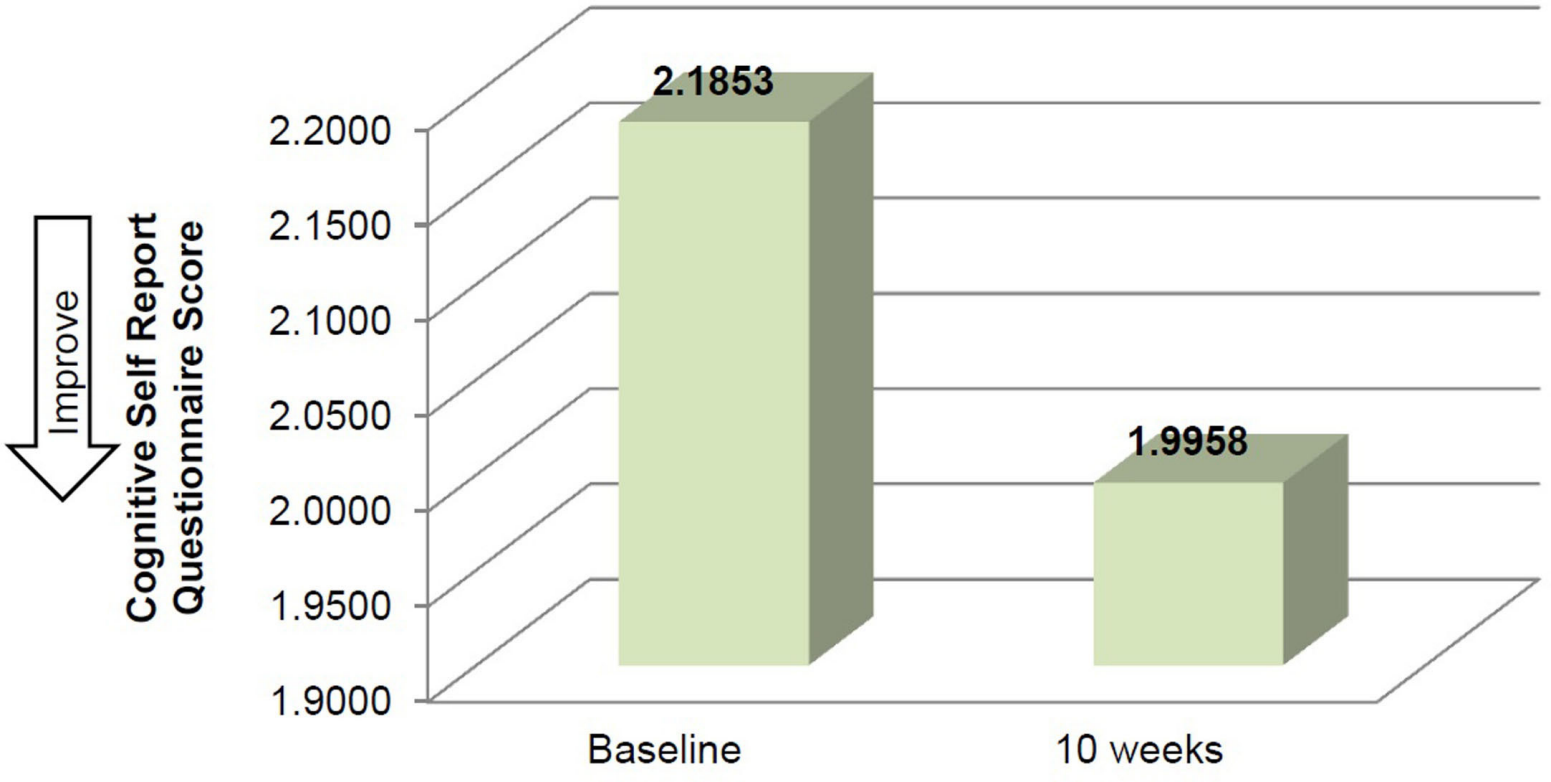
**Change in cognitive self-report questionnaire between baseline and 10 weeks**.

Mean time required to complete TUG improved between baseline and 10 weeks (μ = 25.0 s and μ = 20.8 s, respectively), although this improvement did not reach significance (Table 2). Mean performance on three balance stands (side-by-side μ = 10.30 vs. 10.37 s; partial tandem μ = 9.1 vs. 9.6 s; full tandem μ = 3.5 vs. 5.5 s) also improved at 10 weeks, but did not reach significance. Repeated measures *t*-tests exhibited a significant improvement in depressive symptoms between baseline and 10 weeks [*t*(18) = 2.53, *p* = 0.021] (Figure 2). CSRQ and MMSE improved from baseline (CSRQ: μ = 2.19, SD 0.56; MMSE μ = 21.41, SD 2.90) to follow-up (CSRQ μ = 2.00, SD 0.42; μ = 22.58. SD 3.76), but neither of these improvements were statistically significant [(CSRQ: *t*(17) = 1.63, *p* = 0.122); (MMSE *t*(15) = −0.24, *p* = 0.81)] (Figures 3 and 4).

**Table 2:** **Analytic results for paired-samples *t*-tests**.

Variable	Mean change	SD	SE	*t*	df	Sig.
Timed up and go	0.285	2.660	0.687	0.416	14	0.684
Side-by-side stand	−0.071	0.279	0.066	−1.081	17	0.295
Partial tandem stand	−0.482	3.598	0.933	−0.517	17	0.612
Full tandem stand	−1.836	4.666	1.100	−1.670	17	0.113
Cognitive self-report questionnaire	0.120	0.313	0.074	1.627	17	0.122
Mini mental state exam	−0.125	2.094	0.523	−0.239	15	0.814
Geriatric depression scale	2.263	3.900	0.895	2.530	18	*0.021

*p < 0.05.

Qualitative data revealed that participants had an overall favorable impression of the program. One participant stated “come and enjoy and find that you’re more capable than you might think.” Program staff reported that participants were not only capable of completing the program, but remained engaged and challenged throughout. In a post-program interview, one staff member stated that “individuals in the midst of losing control of their mind found moments during CT that they felt in control again, which appeared to increase confidence and self-worth.”

The feasibility of the program was assessed based upon organization-level factors. Key informant interviews were conducted with the program director and staff delivering the program. Overall, the staff reported that the program was feasible and successful. The program director stated that she “feels it is purposeful programing and effective… overall (participants) enjoyed the program” and noted that when participants “improved confidence in one area, the confidence trickles into other (behaviors)”. The staff delivering the intervention also reported that improvements in participants’ confidence were apparent over the course of the 10 weeks. The program was embedded within the structure of the adult day program and required few additional resources to implement. However, one challenge identified by key informants was facility space. Due to limited space availability, they expressed that it would likely be difficult to identify a quiet space to conduct this intervention on an ongoing basis.

## Discussion

To our knowledge, this is the first study to examine whether cognitively impaired older adults experience improvements in balance following CT. Participants improved in 4 of the 5 balance measures over the study period. Although none of the balance improvements reached significance, these findings are promising given the small sample size.

The primary purpose of this study was to examine the feasibility of conducting a group-based CT intervention among a cohort of cognitively impaired older adults. The program was embedded within an Easter Seals Adult Day Program. Program staff received training to implement the CT program and to collect study measures. Key informant interviews at the conclusion of the program with the program director and staff revealed that with the exception of limited space availability, the program implementation was both feasible and enthusiastically embraced by both staff and participants. Based on the successful implementation of the program within this environment and among this cohort, we conclude that it is feasible to implement a CT program within an adult day program.

Our repeated measures analysis found that balance, depressive symptoms, and global cognition improved in the hypothesized direction following the 10-week CT program; however, depressive symptoms was the only outcome to significantly improve among our cohort of cognitively impaired older adults. This may be the first CT intervention to examine whether cognitively impaired older adults experience improvements in depressive symptoms. During post-program participant debriefing sessions, many expressed having a positive experience with the program and, following an initial period of staff guidance, increased confidence in their ability to complete the CT independently resulting in a sense of cognitive control. Staff also observed an improvement in participant confidence over the 10-week training period. It is possible that the self-reported improved sense of independence and control following the program mediated the significant reduction in depressive symptoms.

We found that mean balance performance on TUG and three of the four balance stands improved across the 10-week training period; however, the post-program improvement in balance was not significant. To date, six studies, including two by Smith-Ray, have shown that CT improves motor tasks, such as balance and walking in older adults (25–28, 37, 38). Four of the studies found that balance significantly improved following CT (25–28), but only the studies by Li et al. and the two Smith-Ray et al. studies used randomized trials and found that participants in the CT arm improved significantly in balance compared to controls. Although the number of studies that have addressed this issue is limited, collectively, the findings indicate that it may be plausible to improve balance, and subsequently fall risk, by using CT. The present study is the first, to our knowledge, to record an improvement in balance following CT among a cohort of cognitively impaired older adults.

This study is not without limitations. First, limited resources were available to conduct this study, and as such, we were unable to collect and analyze data on important covariates, such as polypharmacy/medication regimen, which is known to impact balance. Because we were unable to control for confounding factors, we cannot say conclusively that the significant improvements in depressive symptoms were due to CT rather than another explanatory factor, such as change in social engagement. We were also unable to conduct a cost effectiveness analysis of the intervention; such evidence would provide further support to justify or refute the feasibility of this intervention. Another limitation of the study was the use of a pre-post, within-subjects design with a small number of participants. Larger randomized studies among this population are needed to confirm these findings.

## Conclusion

Our findings support the feasibility of implementing a CT intervention to cognitively impaired older adults within an adult day setting. We found a significant improvement in depressive symptoms post-program in addition to improvements in global cognition that did not reach significance. We also found that balance improved within participants following 10 weeks of CT. These results are in line with findings reported by similar studies and support the hypothesis that CT may be a novel approach to improve balance among older adults. This study not only adds to emerging literature that CT may be a novel and innovative approach to fall prevention among older adults, but is the first to demonstrate this relationship among a cohort of cognitively impaired older adults.

## Author Contributions

RS-R and CI co-led this project as investigators and together developed the research method, analytic plan, identified research site, and trained staff on data collection. Ms. KB assisted with data analysis and manuscript writing.

## Conflict of Interest Statement

The authors declare that the research was conducted in the absence of any commercial or financial relationships that could be construed as a potential conflict of interest.
